# Attitudes toward partner notification of *Chlamydia trachomatis* infection in Shenzhen, China: independent and interactive effects of sociosexual orientation and social support

**DOI:** 10.3389/fpubh.2026.1754680

**Published:** 2026-02-16

**Authors:** Ying Xiong, Weisen Zhao, Yiqiong Wen, Zheng Wu, Jiaming Ye, Jiqiang Xing, Shi Liang, Shuxia Chang

**Affiliations:** 1 Department of Dermatology and Venereology, Shenzhen Longgang Center for Chronic Disease Control, Shenzhen, Guangdong, China; 2 Department of Dermatology, Longgang District People’s Hospital, Shenzhen, China; 3 Department of Biobank, Suzhou Center for Disease Control and Prevention, Suzhou, Jiangsu, China; 4 Division of Radiological Health and Occupational Health, Xiamen Center for Disease Control and Prevention, Xiamen, Fujian, China; 5 Health Management Center, Pingshan General Hospital, Southern Medical University, Shenzhen, Guangdong, China; 6 Health Management Center, Pingshan District Peoples’ Hospital of Shenzhen, Shenzhen, Guangdong, China

**Keywords:** additive interaction, genital *Chlamydia trachomatis* infection, influencing factors, partner notification, perceived social support, sociosexual orientation

## Abstract

**Background:**

Genital *Chlamydia trachomatis* (GCT) infection continues to be a widespread sexually transmitted infection (STI) globally, with China facing challenges in implementing the policy of partner notification (PN). To effectively manage GCT infection, it is crucial to understand the public’s perspectives on PN and identify the psychosocial factors influencing these attitudes. This study aimed to investigate the determinants of willingness toward PN of GCT infection, specifically focusing on the independent and interactive effects of sociosexual orientation and perceived social support among community residents in Longgang District, Shenzhen, China.

**Methods:**

A multistage random sampling approach was employed to collect survey data from 3,453 residents aged 18–60 years in Longgang District, Shenzhen, between November 2024 and February 2025. The revised Sociosexual Orientation Scale (SOI-R) was used to assess sociosexual orientation, while the Perceived Social Support Scale (PSSS) was employed to evaluate social support. Bidirectional stepwise logistic regression was performed to identify key independent factors associated with PN willingness. Subgroup analyses were performed to explore the influences of key demographic variables in different population subgroups on the sociosexual orientation and social support. Additionally, additive interaction analyses were conducted to comprehensively examine the independent and interactive effects of sociosexual orientation and social support on PN willingness.

**Results:**

Overall, 95.83% of participants expressed willingness to notify their partners in the hypothetical scenario of contracting a GCT infection. Factors significantly linked to greater PN willingness included higher education level, non-local household registration, self-rated ordinary or poor economic status, a history of STIs, higher perceived social support, and a more restricted sociosexual orientation. Notably, a positive additive interaction trend was observed between a restricted sociosexual orientation and moderate to high perceived social support, suggesting that their joint effect on PN willingness may exceed the sum of their individual effects.

**Conclusion:**

In Longgang District, Shenzhen, China, people have a relatively high acceptance of PN for GCT infection. Sociosexual orientation and perceived social support were independently associated with attitudes toward PN, and their joint effect may further enhance PN willingness. These findings provide exploratory evidence to inform tailored interventions integrating psychological and social factors to strengthen STI prevention strategies.

## 1 Introduction

Genital *Chlamydia trachomatis* (GCT) infection is one of the most common sexually transmitted infections (STIs) worldwide. Its complications, such as pelvic inflammatory disease, tubal infertility, ectopic pregnancy, and chronic pelvic pain, pose a serious threat to reproductive health and impose significant physical, psychological, and socioeconomic burdens on infected individuals (1). However, GCT infection is often asymptomatic or presents with mild symptoms, particularly among women, allowing it to spread unnoticed and giving rise to the characteristic “silent epidemic” (2). This characteristic highlights the importance of partner notification (PN). PN is the process of identifying, notifying, and encouraging sexual partners to seek testing and treatment, and it serves as a key intervention to break the chain of transmission, prevent reinfection, and reduce complications (3–6). A health economic assessment conducted in the UK demonstrated that PN is a highly cost-effective yet underutilized infection control strategy (7).

The GCT infection has long been recognized as a considerable global public health concern. The World Health Organization estimates for 2020 indicate that roughly 129 million people aged 15–49 years were affected, accounting for nearly one-third of all new cases among the four curable STIs: chlamydia, gonorrhea, trichomoniasis, and syphilis (1). The GCT infection situation in China is similarly concerning. Since 2008, the reported incidence has increased from 32.48 per 100,000 to 55.32 per 100,000 in 2019, with the sharpest rise occurring between 2015 and 2019, when the average annual growth rate reached 10.44% (8, 9). Despite this growing burden, China has not yet developed standardized PN policies specific to GCT infection (10). In addition, empirical research on public perceptions of GCT and the factors influencing attitudes toward PN remains limited.

In recent years, Longgang District in Shenzhen, Guangdong Province, has spearheaded PN practices for GCT infection in China. This pioneering initiative not only provides valuable experience for national-scale prevention and control efforts but also highlights the urgent need for localized empirical evidence. Specifically, there is a pressing need to systematically explore the key factors that enable or hinder PN behavior within China’s unique sociocultural framework.

Currently, PN efforts have been primarily applied in the prevention and control of HIV and syphilis, whereas strategies for PN related to GCT infection remain relatively limited, particularly within the Chinese context (10, 11). PN is a multifaceted health behavior decision influenced by various factors extending beyond knowledge and attitudes to deeper individual traits and environmental resources. Hence, this study focused on sociosexual orientation and perceived social support, representing individual propensities for non-committed sexual relationships and psychosocial resources during stressful times, respectively (12–14). By examining their independent and potential interactive effects on PN attitudes in the context of GCT infection, this study aimed to clarify how these factors contribute to sexual health decision-making. Insights from this work may help inform more targeted and context-appropriate PN strategies within China’s diverse sociocultural setting.

In summary, taking Longgang District as the study object, this study aimed to systematically explore the key factors influencing attitudes toward PN of GCT infection among the general Chinese population. In particular, this study focused on the interaction between sociosexual orientation and perceived social support to address the existing gap in context-specific evidence. The study findings are expected to provide scientific support for the design of more precise and effective intervention strategies and hold important public health implications for strengthening the prevention and control of STIs.

## 2 Materials and methods

### 2.1 Sample and sampling

The inclusion criteria for the research sample were as follows: individuals aged 18–60 years; residents of Longgang District, Shenzhen, for at least six consecutive months; and able to complete the survey independently. This study was a cross-sectional study of the general population. However, due to the lack of reference data on the PN willingness rate (P) in the general population, we chose to select *p* = 60% as the parameter for sample size calculation, based on the following data sources: (1) a 31.6% PN rate among STI patients in China (from a 2012 systematic review) (10); (2) an 87.31% PN willingness rate among female patients at a STI clinic in Shenzhen (from a 2020 cross-sectional study) (15); (3) a 39.1% PN willingness rate among GCT-infected individuals in Longgang District, Shenzhen (from data on gonorrhea and the GCT infection partner management program of the Longgang Centre for Chronic Disease Control).

The “Confidence Intervals for One Proportion” module in PASS 2021 software was employed for sample size determination, and the Exact (Clopper-Pearson) formula was applied to calculate the required sample size. The significance level (α) was set at 0.01, the permissible error (δ) (two-sided) was set at 0.05, the estimated PN willingness rate was set at 60%, and the sample loss rate was set at 20%. Based on these parameters, the required sample size was 3,230.

From November 2024 to February 2025, a cluster random multistage sampling strategy was employed in 11 sub-districts of Longgang District. In the first stage, based on regional accessibility, Longgang District was selected as the sample collection area. In the second stage, two communities were randomly selected in each sub-district (a total of 22 communities). In the third stage, three residential groups were randomly selected in each selected community (a total of 66 residential groups). In the fourth stage, at least 50 participants were randomly selected in each selected residential group or compound. Selected participants were invited to complete an online survey (a total of 3,300 participants).

In order to guarantee data quality and adhere to the inclusion criteria, surveys with abnormal data, short completion times (less than 180 s), or logical errors, and responses from individuals younger than 18 years or older than 60 years, were excluded. After screening, 3,453 valid surveys were included in the analyses. All participants provided informed consent prior to participation. This study was conducted in accordance with the principles of the Helsinki Declaration.

### 2.2 Survey and measurement tools

Data for this study were collected with the use of a self-administered structured survey. The survey included the following main sections:

Sociodemographic characteristics, including age, gender, education level, marital status, registered place of residence, economic status, history of STIs, and discrimination against patients with STIs.

Participants’ attitudes toward PN were measured with the question, “If you were hypothetically infected with GCT, would you be willing to notify your sexual partner(s) to undergo GCT screening?” Responses included “willing” or “unwilling.”

Sociosexual orientation, referring to the inclination for non-committal sexual relationships, was assessed using the Chinese version of the Sociosexual Orientation Inventory–Revised (SOI-R). This scale consists of nine items that evaluate three dimensions—sociosexual behavior, attitude, and desire—using a nine-point Likert scale. The total score ranges from 9 to 81, with higher scores indicating a more unrestricted sociosexual orientation, while lower scores reflect a more restricted orientation (16). Participants were further divided into two groups (Restricted vs. Unrestricted) according to the median score of the whole sample (17). The SOI-R has demonstrated good reliability and validity among Chinese populations (12), with a Cronbach’s *α* of 0.83 in this study.

Perceived social support was measured using the Perceived Social Support Scale (PSSS). This scale assesses the extent of support participants might perceive from family, friends, and others in the hypothetical situation of GCT infection (18, 19). The PSSS includes 12 items rated on a seven-point Likert scale ranging from 1 (“strongly disagree”) to 7 (“strongly agree”). The total score ranges from 12 to 84, with higher scores indicating greater perceived social support. Scores between 12 and 36 indicate low support, 37–60 indicate moderate support, and 61–84 indicate high support (20). In this study, the Cronbach’s *α* of the PSSS was 0.97, indicating excellent internal consistency.

### 2.3 Quality control

To ensure the reliability of the study and the validity of the data, rigorous quality control measures were implemented throughout the study design, data collection, and data cleaning:

Study design phase: Prior to the formal survey, a small-scale pilot study was conducted to evaluate the comprehensibility, completion time, and clarity of the survey items. Based on participant feedback, minor revisions were made to the wording of certain items to ensure their appropriateness and intelligibility within the Chinese cultural context. All researchers involved in the study received standardized and uniform training.

Data collection process: Before completing the survey, all participants were required to read and electronically sign an online informed consent form. The survey system automatically recorded the completion time of each questionnaire. Investigators adhered to standardized survey procedures and interacted with participants in a polite and respectful manner.

Data management: All collected data were immediately backed up after export to prevent data loss. Responses with apparent logical inconsistencies were flagged and manually reviewed to ensure data quality.

### 2.4 Statistical analysis

Descriptive analyses were performed to summarize participants’ general characteristics. Normally distributed data are expressed as *x̄* ± *s* and were compared between groups using analysis of variance (ANOVA). Non-normally distributed data are presented as M (Q₁, Q₃) and were compared using the rank-sum test. Categorical variables are described as frequencies (percentages), and intergroup differences were assessed using the *χ*^2^ test.

A logistic regression model was used to identify factors independently associated with the willingness to engage in PN. The results are expressed as odds ratios (ORs) and adjusted odds ratios (AORs) with corresponding 95% confidence intervals (95% CIs). Subgroup analyses were further conducted based on key demographic variables to examine the stability of the main variables (sociosexual orientation and perceived social support) across different population subgroups.

Finally, an interaction term between sociosexual orientation and social support was constructed and incorporated into the logistic regression model to test the core hypothesis of the interaction effect. The statistical significance of the interaction term (*p* < 0.05) was used to assess for a multiplicative interaction. In addition, the presence of an additive interaction was evaluated using three standard epidemiological measures—relative excess risk due to interaction (RERI), attributable proportion due to interaction (AP), and synergy index (SI) (21)—because effect modification on the absolute (additive) scale is often more informative for public health–oriented interpretation (22). For additive interaction estimation, sociosexual orientation was categorized as restricted versus unrestricted and perceived social support as low, moderate, or high, using the low perceived support + unrestricted sociosexual orientation group as the reference category. RERI, AP, and SI were computed on the ORs scale, and 95% confidence intervals were obtained via nonparametric bootstrapping with 1,000 resamples. These indices were derived from ORs estimated by logistic regression and are, therefore, interpreted on the OR scale. An additive interaction was considered absent if the 95% CI for RERI and AP included 0 and the 95% CI for SI included 1 (21).

All statistical analyses were performed using Excel software (version 2021) and R software (version 4.1.2). A two-sided *p* < 0.05 was considered statistically significant.

## 3 Results

### 3.1 Demographic data

A total of 3,453 valid surveys were analyzed. The vast majority of participants (95.83%) expressed a willingness to inform their sexual partners in the event of a hypothetical GCT infection. The participants were primarily female (57.11%), married (58.12%), non-local residents (71.33%), and had a college degree or above (42.92%). Most participants had no history of sexually transmitted diseases (97.45%), held no discriminatory attitudes toward patients with STIs (65.74%), and self-assessed their economic status as poor (63.3%). The median age, SOI-R score, and PSSS score were 36.00 years (IQR: 28.00–43.00), 17.00 (IQR: 11.00–22.00), and 48.00 (IQR: 42.00–62.00), respectively (Table 1). There were statistically significant differences in PN willingness across participants with varying educational levels, economic statuses, STI histories, discriminatory attitudes toward patients with STIs, sociosexual orientations, and perceived social support (Table 1).

**Table 1:** Participant demographic information.

Characteristic	Overall	Unwilling-to-PN	Willing-to-PN	Test statistics	*p*-value
Total (%)	3,453 (100.00%)	144 (100.00%)	3,309 (100.00%)		
Gender (%)				0.812	0.368
Female	1,972 (57.11%)	77 (53.47%)	1,895 (57.27%)		
Male	1,481 (42.89%)	67 (46.53%)	1,414 (42.73%)		
Age group (%)				5.083	0.079
≤30 years	1,110 (32.15%)	34 (23.61%)	1,076 (32.52%)		
31–40 years	1,219 (35.30%)	56 (38.89%)	1,163 (35.15%)		
>40 years	1,124 (32.55%)	54 (37.50%)	1,070 (32.34%)		
Education (%)				49.439	<0.001
Junior high school and below	1,086 (31.45%)	80 (55.56%)	1,006 (30.40%)		
Senior high school	885 (25.63%)	38 (26.39%)	847 (25.60%)		
College and above	1,482 (42.92%)	26 (18.06%)	1,456 (44.00%)		
Marital status (%)					0.435
Married	2,007 (58.12%)	84 (58.33%)	1,923 (58.11%)		
In a relationship	284 (8.22%)	7 (4.86%)	277 (8.37%)		
Single	1,048 (30.35%)	49 (34.03%)	999 (30.19%)		
Divorced/widowed	114 (3.30%)	4 (2.78%)	110 (3.32%)		
Place of residence (%)				0.99	0.32
Local	990 (28.67%)	36 (25.00%)	954 (28.83%)		
Non-local	2,463 (71.33%)	108 (75.00%)	2,355 (71.17%)		
Self-assessment of economic (%)				10.629	0.005
Better	186 (5.39%)	12 (8.33%)	174 (5.26%)		
Ordinary	1,070 (30.99%)	28 (19.44%)	1,042 (31.49%)		
Poor	2,197 (63.63%)	104 (72.22%)	2,093 (63.25%)		
Past STIs (%)				13.612	<0.001
Yes	88 (2.55%)	11 (7.64%)	77 (2.33%)		
No	3,365 (97.45%)	133 (92.36%)	3,232 (97.67%)		
Discrimination against people with STIs (%)				1.291	0.256
Non-discrimination	2,270 (65.74%)	101 (70.14%)	2,169 (65.55%)		
Discrimination	1,183 (34.26%)	43 (29.86%)	1,140 (34.45%)		
Perceived social support level (%)				41.63	<0.001
Low	710 (20.56%)	60 (41.67%)	650 (19.64%)		
Moderate	1,829 (52.97%)	60 (41.67%)	1,769 (53.46%)		
High	914 (26.47%)	24 (16.67%)	890 (26.90%)		
Sociosexual orientation (%)				26.782	<0.001
Unrestricted	1,717 (49.72%)	102 (70.83%)	1,615 (48.81%)		
Restricted	1,736 (50.28%)	42 (29.17%)	1,694 (51.19%)		
Age [*M* (Q1, Q3)]	36.00 (28.00, 43.00)	38.00 (31.00, 46.00)	36.00 (28.00, 43.00)	266924.5	0.014
PSSS^1^ total score [*M* (Q1, Q3)]	48.00 (42.00, 62.00)	45.50 (12.00, 51.00)	48.00 (44.00, 62.00)	169990.5	<0.001
SOI-R^2^ total score [*M* (Q1, Q3)]	17.00 (11.00, 22.00)	21.00 (17.00, 29.00)	17.00 (11.00, 21.00)	330,941	<0.001

^1^PSSS: Perceived Social Support Scale.

^2^SOI-R: revised Sociosexual Orientation Scale.

### 3.2 Significant factors associated with PN willingness

After including all 10 variables in the model, multivariate bidirectional stepwise logistic regression analysis identified several significant factors associated with willingness to engage in PN. Higher education levels (senior high school: AOR = 1.70, 95% CI = 1.13–2.57; college or above: AOR = 4.75, 95% CI = 2.81–8.04), non-local household registration (AOR = 1.62, 95% CI = 1.04–2.53), self-rated ordinary (AOR = 2.27, 95% CI = 1.10–4.69) or poor economic status (AOR = 2.12, 95% CI = 1.09–4.11), a history of STIs (AOR = 2.49, 95% CI = 1.25–4.98), higher levels of perceived social support (moderate: AOR = 2.32, 95% CI = 1.59–3.39; high: AOR = 2.76, 95% CI = 1.68–4.54), and a more restricted sociosexual orientation (AOR = 2.15, 95% CI = 1.48–3.12) were all positively associated with PN willingness (Supplementary Table 1). In contrast, gender, age, marital status, and discriminatory attitudes toward patients with STIs were not significantly associated with PN willingness.

### 3.3 Associations of sociosexual orientation and perceived social support with PN willingness

We further examined the promoting effects of sociosexual orientation and perceived social support on PN willingness. In the unadjusted model (Model 1), a more restricted sociosexual orientation (OR = 2.55, 95% CI = 1.77–3.67) and higher levels of perceived social support (moderate: OR = 2.72, 95% CI = 1.88–3.94; high: OR = 3.43, 95% CI = 2.11–5.55) were both significantly associated with PN willingness (Table 2). When analyzed as continuous variables, for every one-point increase in SOI-R and PSSS scores, PN willingness decreased by 6% and increased by 3%, respectively (Table 2). After gradually adjusting for gender and age (Model 2) and other covariates (Model 3), these associations remained statistically significant, with stable effect sizes, indicating that a more restricted sociosexual orientation and higher levels of perceived social support were consistently associated with willingness to engage in PN (Table 2).

**Table 2:** Association between sociosexual orientation, perceived social support, and partner notification.

Characteristic	Model 1		Model 2		Model 3	
	OR (95% CI)	*p*-value	OR (95% CI)	*p*-value	OR (95% CI)	*p*-value
Sociosexual orientation						
Unrestricted	1.00		1.00		1.00	
Restricted	2.55 (1.77–3.67)	<0.001	2.47 (1.72 to 3.61)	<0.001	2.17 (1.50 to 3.19)	<0.001
SOIR^1^ total score	0.94 (0.92–0.95)	<0.001	0.93 (0.92 to 0.95)	<0.001	0.93 (0.92 to 0.95)	<0.001
Perceived social support level						
Low	1.00		1.00		1.00	
Moderate	2.72 (1.88–3.94)	<0.001	2.63 (1.81 to 3.81)	<0.001	2.43 (1.65 to 3.58)	<0.001
High	3.42 (2.11–5.55)	<0.001	3.31 (2.07 to 5.48)	<0.001	3.29 (1.99 to 5.63)	<0.001
PSSS^2^ total score	1.03 (1.02–1.03)	<0.001	1.02 (1.02 to 1.03)	<0.001	1.02 (1.02 to 1.03)	<0.001

Model 1: no covariates were adjusted.

Model 2: adjust for: Gender + Age.

Model 3: adjust for: Gender + Age + Education + Marital status + Place of residence + Self-assessment of economic status + Past STIs + Discrimination against people with STIs.

^1^PSSS: perceived Social Support Scale.

^2^SOI-R: revised Sociosexual Orientation Scale.

### 3.4 Subgroup analysis

**Figure 1:**
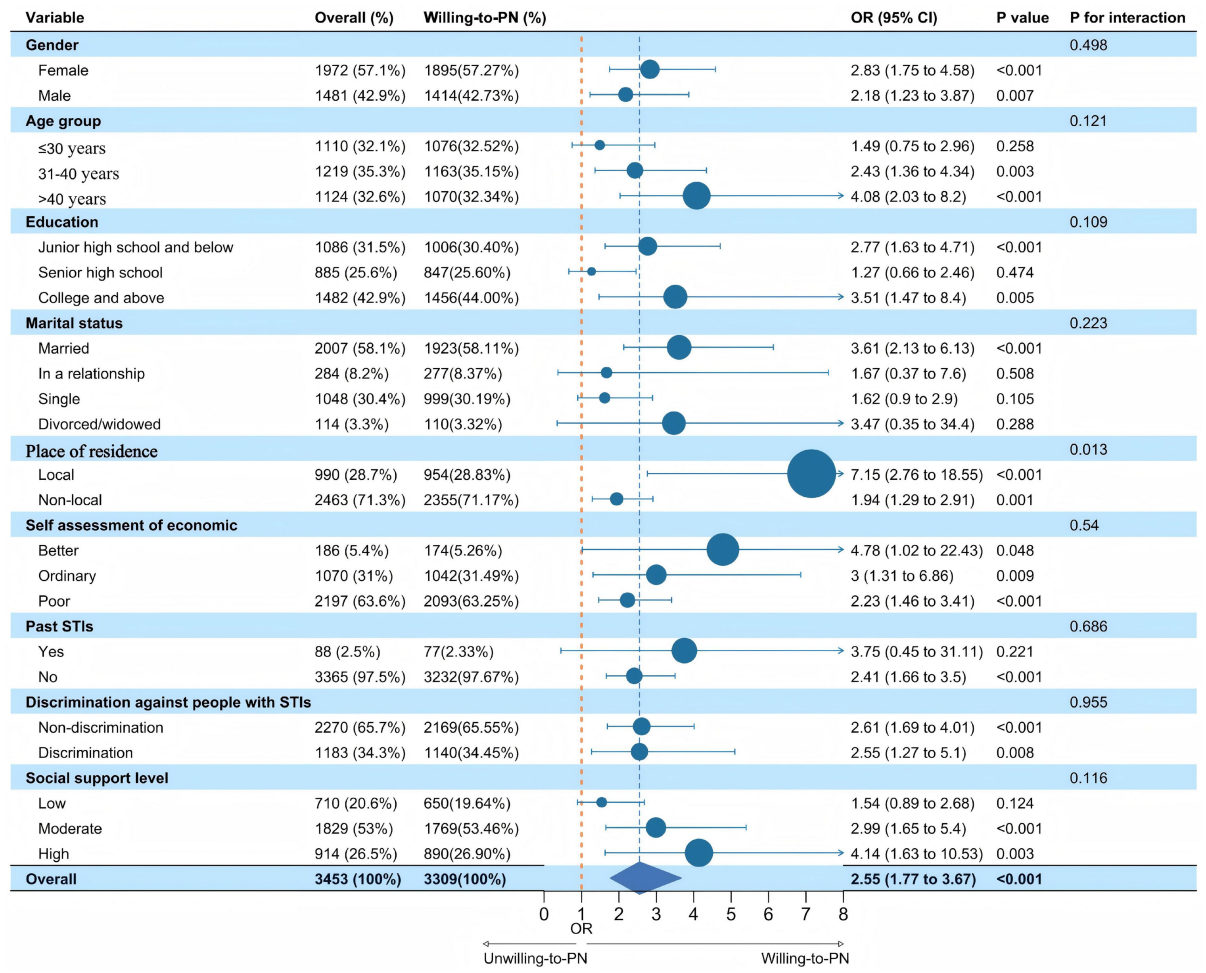
Forest plot showing the association between sociosexual orientation and PN willingness across demographic subgroups.

Both a restricted sociosexual orientation and higher levels of perceived social support were significantly associated with stronger willingness to engage in PN, and these associations remained generally robust across all subgroups. Among participants with local household registration, the effect of sociosexual orientation on PN willingness (OR = 7.15, 95% CI: 2.76–18.55) was substantially stronger than that observed among non-local residents (OR = 1.94, 95% CI: 1.29–2.91) (Figure 1).

The effect of perceived social support on PN willingness showed heterogeneity across subgroups defined by education level and household registration. Specifically, as the level of perceived social support increased, PN willingness strengthened among participants with education levels of junior high school or below, as well as among those with college education or above, while participants with high school education showed the highest PN willingness at a moderate level of social support. Among non-local residents, PN willingness significantly increased at both moderate and high levels of perceived social support, whereas no significant association was observed among local residents (Supplementary Figure 1).

### 3.5 Interaction analysis

**Figure 2:**
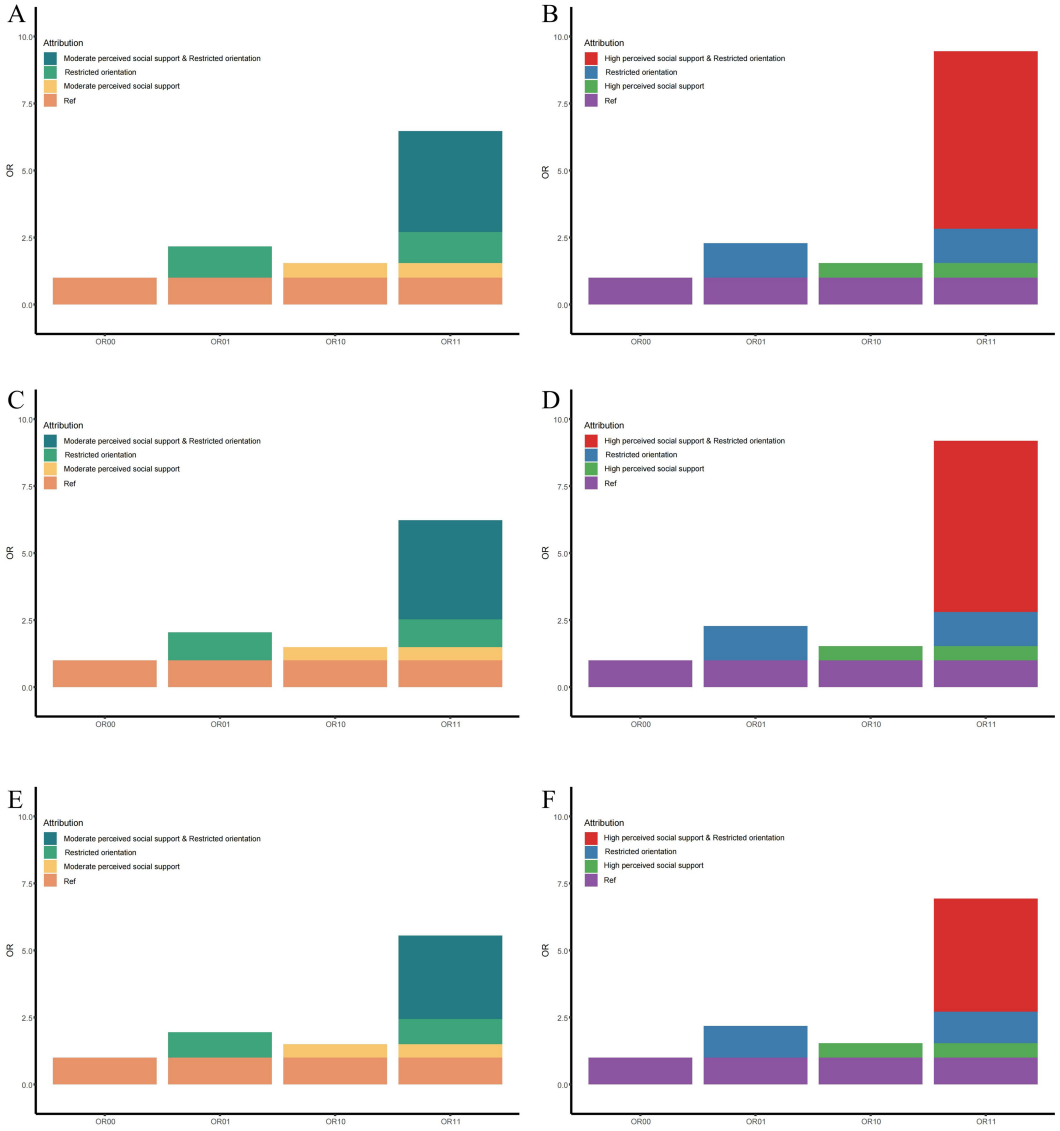
Additive interaction between sociosexual orientation and perceived social support on PN willingness. This figure illustrates the additive interaction between sociosexual orientation and perceived social support on PN willingness. Panels **(A,C,E)** present the interaction between restricted sociosexual orientation and moderate perceived social support, whereas panels **(B,D,F)** present the interaction between restricted sociosexual orientation and high perceived social support. Model adjustment differs by panel: **(A,B)** show crude estimates (no covariates adjusted); **(C,D)** adjust for gender and age; and **(E,F)** further adjust for gender, age, education, marital status, place of residence, self-assessment of economic status, past STIs, and discrimination against people with STIs. ORs are shown for four exposure combinations: OR00, low perceived social support + unrestricted sociosexual orientation (reference group, OR = 1.0); OR01, low perceived social support + restricted sociosexual orientation; OR10, moderate **(A,C,E)** or high **(B,D,F)** perceived social support + unrestricted sociosexual orientation; OR11, moderate **(A,C,E)** or high **(B,D,F)** perceived social support + restricted sociosexual orientation.

The interaction analysis revealed that a restricted sociosexual orientation combined with moderate or high levels of perceived social support was associated with higher willingness to engage in PN compared with the reference group (unrestricted sociosexual orientation and low perceived social support) (Table 3 and Figure 2). On the multiplicative scale, the interaction terms showed a positive trend but did not reach statistical significance across the models. On the additive scale, all interaction indicators demonstrated consistently positive estimates.

**Table 3:** Interaction effect of sociosexual orientation and perceived social support on partner notification.

Model	Measure	Restricted sociosexual orientation and moderate social support	Restricted sociosexual orientation and high social support
Model 1	OR (95%CI)	6.3 (3.42, 11.6)	9.19 (3.84, 21.96)
	Multiplicative scale (95%CI)	1.99 (0.88, 4.48)	2.75 (0.93, 8.14)
	Additive scale (95% CI)		
	RERI^1^	3.69 (0.99, 10.15)	6.46 (1.61, 27.65)
	AP^2^	0.59 (0.22, 0.81)	0.70 (0.34, 0.92)
	SI^3^	3.30 (1.37, 14.52)	4.74 (1.54, 28.33)
Model 2	OR (95%CI)	6.22 (3.37, 11.49)	9.18 (3.83, 22.04)
	Multiplicative scale (95%CI)	2.05 (0.91, 4.62)	2.65 (0.89, 7.85)
	Additive scale (95% CI)		
	RERI	3.69 (1.03, 9.53)	6.38 (1.32, 30.04)
	AP	0.59 (0.24, 0.8)	0.69 (0.26, 0.91)
	SI	3.42 (1.41, 14.11)	4.54 (1.44, 32.82)
Model 3	OR (95%CI)	5.55 (2.97, 10.37)	6.93 (2.84, 16.89)
	Multiplicative scale (95%CI)	1.91 (0.84, 4.35)	2.07 (0.69, 6.24)
	Additive scale (95% CI)		
	RERI	3.11 (0.73, 8.59)	4.21 (0.08, 22.65)
	AP	0.56 (0.2, 0.79)	0.61 (0.02, 0.89)
	SI	3.16 (1.22, 15.14)	3.46 (0.94, 25.66)

Model 1: no covariates were adjusted.

Model 2: adjust for: Gender + Age.

Model 3: adjust for: Gender + Age +  Education + Marital status + Place of residence + Self-assessment of economic status + Past STIs + Discrimination against people with STIs.

^1^RERI: relative excess risk due to interaction.

^2^AP: attributable proportion due to interaction.

^3^SI: synergy index.

Across Models 1, 2, and 3, the combination of restricted sociosexual orientation and moderate perceived social support yielded statistically significant estimates for RERI, AP, and SI. Furthermore, when restricted sociosexual orientation was combined with high perceived social support, the magnitude of the additive interaction indicators increased, indicating a stronger additive interaction trend (Table 3).

## 4 Discussion

In this study of the general population aged 18–60 years in Longgang District, when hypothetically infected with GCT, 95.83% of participants expressed a willingness to engage in PN. This proportion is remarkably high, suggesting that the public generally accepts the PN policy and that societal awareness of health responsibility and risk communication is gradually maturing. This estimate is higher than the PN willingness reported among women attending reproductive health and STI clinics in Shenzhen (87.31%) and is also at the upper end of the PN intention levels reported in some international studies (>70%) (15, 23–25). However, we note that willingness measured hypothetically may not translate into actual PN behavior. The high estimate may also reflect survey framing and anonymous online administration, which can increase socially desirable responding. In addition, Longgang District has been a pioneering area for GCT-related partner management practices, which may increase public awareness and normative endorsement of PN. Importantly, prior studies in China have reported substantially lower PN uptake when measured as actual behavior among STI patients, while higher willingness has been observed in some clinic-based samples (10, 15). Therefore, heterogeneity across studies is expected due to differences in study populations (community vs. clinic), outcome definitions (hypothetical willingness vs. actual PN uptake), and local service context. Taken together, our findings complement prior epidemiological evidence that largely focuses on clinic-based populations and observed PN uptake by providing population-based, psychosocially informed estimates of PN willingness. Nonetheless, these findings underscore the need for future studies linking these psychosocial profiles to real-world PN behaviors and partner outcomes.

Further analysis revealed that higher education levels, non-local household registration, self-rated ordinary or poor economic status, a history of STIs, higher perceived social support, and a more restricted sociosexual orientation were all significantly associated with higher PN willingness. These findings align with the core assumptions of health belief models and social cognitive theory—that health decisions are shaped by cognition, experience, and psychological traits (26, 27). Notably, this study is the first to reveal the independent and interactive effects of sociosexual orientation and perceived social support on PN willingness in the condition of hypothetical GCT infection, and the findings indicate a consistent positive additive interaction trend between the two factors, providing new insights into the social-psychological mechanisms underlying health behaviors.

Consistent with previous research, education level was found to be a crucial factor in promoting PN willingness (15, 28, 29). Individuals with higher education levels typically have better health literacy, allowing them to better understand the risks of disease transmission (30). They also tend to have stronger self-efficacy, which increases confidence in navigating sensitive communication situations (31). Furthermore, higher education enhances an individual’s social cognition and emotional regulation abilities (32), helping to reduce feelings of shame and self-blame associated with STIs, and thereby making them more likely to engage in responsible PN behaviors.

An intriguing finding of this study is that participants with local household registration and higher economic status showed lower PN willingness. We speculate that this group of individuals might possess more intimate social connections locally and may be more concerned about their own social reputation in the community (33, 34). Therefore, they may be more sensitive to the stigma and privacy risks associated with GCT infection. They may be worried about losing “face” or damaging their social image, thus leading to psychological withdrawal (35). This finding is in sharp contrast to two other studies based on patients in STI clinics, which found that the higher the economic level, the stronger the willingness for PN (15, 36). This discrepancy may be attributed to differences in the populations studied: clinic patients may have a stronger sense of health threat, while the general population in this study may be more concerned about maintaining their social image. Additionally, individuals with a history of STIs were more willing to notify their partners, which may reflect their heightened risk awareness and increased self-efficacy after experiencing infection, further demonstrating the role of experiential learning in health decision-making.

This study utilized the standardized PSSS to objectively and systematically evaluate the facilitating effect of social support on PN willingness, confirming the critical role of social support—as a modifiable external resource—in promoting sexual health. Although some previous studies did not utilize standardized measurement tools, their conclusions similarly indicated a positive association between social support and PN willingness (6, 13, 37), collectively reinforcing the universal importance of social support in enhancing willingness to engage in PN.

This study used the well-established SOI-R to assess participants’ tendencies toward non-committed relationships. The results showed that individuals with a more restricted sociosexual orientation had a higher willingness to engage in PN. This finding may reflect a stronger sense of responsibility and risk awareness in sexual health decision-making among this group. This result is consistent with multiple lines of indirect evidence: previous studies have shown that individuals with more casual sexual relationships or multiple sexual partners usually have a lower likelihood of engaging in PN (24, 27, 38, 39). Furthermore, groups with more unrestricted sociosexual orientations often exhibit higher rates of STIs and higher incidences of high-risk sexual behaviors (40–44). These findings suggest that sociosexual orientation may influence individual health awareness, which, in turn, affects health behaviors and outcomes. This phenomenon highlights the importance of considering individual sociosexual orientation differences when designing sexual health promotion and intervention strategies.

The interaction analysis in this study suggested a positive joint effect of sociosexual orientation and perceived social support on willingness to engage in PN. Although the multiplicative interaction between sociosexual orientation and perceived social support did not reach statistical significance, consistently positive additive interaction indices (RERI, AP, and SI) suggest that their joint effect on PN willingness may exceed the sum of their individual effects. Given the public health relevance of additive interactions for identifying priority subgroups (22), these findings should be interpreted as suggestive rather than definitive evidence of a positive additive interaction. From a public health perspective, this interaction pattern supports an “individual traits + psychosocial resources” framework for understanding PN behaviors and suggests the need for a stratified, stepped-support approach to PN implementation. For individuals reporting lower perceived support—particularly those with more unrestricted sociosexual orientations, who may face higher partner turnover and greater disclosure concerns—PN services could prioritize privacy-preserving, low-burden options such as provider-assisted notification, anonymous/digital notification tools, and standardized message templates. In contrast, among individuals with a more restricted sociosexual orientation, strengthening psychosocial resources (e.g., brief counseling, peer/family support, and stigma-reduction messaging) may further increase PN acceptance and follow-through. Programmatically, brief screening of perceived support and anticipated communication barriers at diagnosis could help match clients to appropriate PN options and allocate counseling and partner services more efficiently. At the policy level, these findings support the integration of psychosocial screening and tiered PN options (self-notification, provider-assisted notification, and digital/anonymous notification) into routine chlamydia case management, alongside confidentiality safeguards and staff training to promote stigma-sensitive partner communication.

This study has several strengths. First, the use of a large-scale community sample and a multistage random sampling design ensured the representativeness and reliability of the results. Second, the core variables were measured using internationally recognized standardized scales (SOI-R and PSSS), ensuring the scientific rigor and comparability of the data. Third, this study is the first to provide population-based evidence suggestive of a positive additive interaction between sociosexual orientation and social support in a community-based sample from Shenzhen (Longgang District), broadening the theoretical perspective in sexual health behavior research. Finally, this study focused on general community residents rather than clinical or high-risk groups, filling a gap in our understanding of PN attitudes related to GCT infection among ordinary residents in China. Accordingly, the findings provide practical insights for public health interventions.

However, there are certain limitations of this study that should be acknowledged. Firstly, the cross-sectional design of this study limits causal inferences. However, given the scarcity of population-based evidence related to PN attitudes toward GCT infection in China, this study was intentionally designed as an exploratory investigation to identify key psychosocial correlates and potential interaction patterns. Accordingly, these findings should be interpreted as hypothesis-generating, providing a foundation for future longitudinal and interventional studies aimed at causal validation.

Secondly, PN willingness was assessed using a hypothetical scenario rather than observed behavior and may, therefore, be overestimated due to social desirability and the intention–behavior gap. In real-world settings, PN can be constrained by stigma, privacy concerns, and relationship factors (6); accordingly, reported willingness should be interpreted cautiously and not conflated with actual PN uptake.

Thirdly, the study sample was primarily drawn from the population of urban residents in Longgang District, a highly urbanized and economically developed area in Shenzhen, China. This population is characterized by a substantial migrant population and relatively good access to STI-related public health services (45). In the Chinese context, pronounced urban–rural disparities persist in terms of health literacy, availability of sexual health services, and implementation capacity of STI prevention programs (46, 47). Moreover, sociocultural factors such as collectivist norms, concerns about “face” (mianzi), family reputation, and stigma surrounding STIs may shape partner communication and disclosure decisions differently across regions (48–50). Perceptions and expectations regarding communication about sexual health issues, stigma levels, and anticipated social consequences of disclosure may, therefore, differ substantially in rural areas or less-developed regions with distinct sociocultural contexts and healthcare infrastructures. Thus, caution is warranted when extrapolating our estimates—particularly the high PN willingness observed—to settings characterized by lower health literacy, more limited service availability, weaker local STI management systems, or stronger community-level stigma. Future research should adopt multi-site designs across diverse regions of China, including rural and under-resourced areas, to assess the robustness and contextual variability of these psychosocial associations.

Finally, although multiple covariates were adjusted for in the analysis, unmeasured factors such as sexual health knowledge and access to medical services may still have contributed to residual confounding. In addition, the binary measurement of PN willingness may not have captured the intensity of willingness or underlying psychological processes. Future studies should consider incorporating more granular outcome measures (e.g., Likert-scale assessments), collecting behavioral PN outcomes (e.g., verified partner contact, testing uptake, or treatment), and adopting sensitivity analyses or longitudinal designs to better capture real-world PN implementation and further validate these findings. In addition, the very high prevalence of PN willingness observed in this study may introduce a ceiling effect and potentially amplify ORs; therefore, alternative modeling approaches estimating risk ratios or marginal effects may be considered in future research.

## 5 Conclusion

In this community-based sample from Longgang District, Shenzhen, hypothetical willingness to engage in PN for GCT infection was high. Multivariate analysis demonstrated that higher education levels, non-local household registration, self-rated average or poor economic status, a history of STIs, higher social support, and a more restricted sociosexual orientation were significantly associated with higher PN willingness. A positive additive interaction was observed between sociosexual orientation and social support. Specifically, in situations with higher social support, individuals with a restricted sociosexual orientation tended to show a stronger willingness to engage in PN. These findings emphasize the critical role of socio-psychological factors in health communication behaviors and provide important theoretical and practical references for PN intervention strategies and sexual health promotion.

## Data Availability

The raw data supporting the conclusions of this article will be made available by the authors, without undue reservation.
