# Supplementary material for: Attitudes toward partner notification of Chlamydia trachomatis infection in Shenzhen, China: independent and interactive effects of sociosexual orientation and social support
Source: Front Public Health. 2026 Feb 16;14:1754680. doi: 10.3389/fpubh.2026.1754680 (PMC12950760; doi:10.3389/fpubh.2026.1754680)
Supplement: Supplementary file 1 [file Supplementary_file_1.docx]

Supplementary Material

# Supplementary Tables and Figures

## Supplementary Tables

**Supplementary Tables 1.** Univariate and multivariate analyses of factors associated with PN willingness.

| **Characteristic** | **Unwilling-to-PN *n(%)*** | **Willing-to-PN *n(%)*** | **OR (95%CI, *p-value*)** | **AOR (95%CI, *p-value*)** |
| --- | --- | --- | --- | --- |
| **Gender** |  |  |  |  |
| Female | 77(53.47%) | 1,895(57.27%) |  |  |
| Male | 67(46.53%) | 1,414(42.73%) | 0.86 (0.61-1.20, p=.368) |  |
| **Age group** |  |  |  |  |
| ≤30 years | 34(23.61%) | 1,076(32.52%) |  |  |
| 31-40 years | 56(38.89%) | 1,163(35.15%) | 0.66 (0.43-1.01, p=.057) |  |
| >40 years | 54(37.50%) | 1,070(32.34%) | 0.63 (0.40-0.97, p=.036) |  |
| **Education** |  |  |  |  |
| Junior high school and below | 80(55.56%) | 1,006(30.40%) |  |  |
| Senior high school | 38(26.39%) | 847(25.60%) | 1.77 (1.19-2.64, p=.005) | 1.70 (1.13-2.57, p=.011) |
| College and above | 26(18.06%) | 1,456(44.00%) | 4.45 (2.84-6.98, p<.001) | 4.75 (2.81-8.04, p<.001) |
| **Marital status** |  |  |  |  |
| Married | 84(58.33%) | 1,923(58.11%) |  |  |
| In a relationship | 7(4.86%) | 277(8.37%) | 1.73 (0.79-3.78, p=.170) |  |
| Single | 49(34.03%) | 999(30.19%) | 0.89 (0.62-1.28, p=.529) |  |
| Divorced/widowed | 4(2.78%) | 110(3.32%) | 1.20 (0.43-3.34, p=.725) |  |
| **Place of residence** |  |  |  |  |
| Local | 36(25.00%) | 954(28.83%) |  |  |
| Non-local | 108(75.00%) | 2,355(71.17%) | 0.82 (0.56-1.21, p=.320) | 1.62 (1.04-2.53, p=.031) |
| **Self-assessment of economic** |  |  |  |  |
| Better | 12(8.33%) | 174(5.26%) |  |  |
| Ordinary | 28(19.44%) | 1,042(31.49%) | 2.57 (1.28-5.14, p=.008) | 2.27 (1.10-4.69, p=.026) |
| Poor | 104(72.22%) | 2,093(63.25%) | 1.39 (0.75-2.57, p=.298) | 2.12 (1.09-4.11, p=.027) |
| **Past STIs** |  |  |  |  |
| Yes | 11(7.64%) | 77(2.33%) |  |  |
| No | 133 (92.36%) | 3232(97.67%) | 3.47 (1.80-6.68, p<.001) | 2.49 (1.25-4.98, p=.009) |
| **Discrimination against people with STIs** |  |  |  |  |
| Non-discrimination | 101(70.14%) | 2,169(65.55%) |  |  |
| Discrimination | 43(29.86%) | 1,140(34.45%) | 1.23 (0.86-1.78, p=.257) | 1.38 (0.95-2.01, p=.095) |
| **Perceived social support level** |  |  |  |  |
| Low | 60(41.67%) | 650(19.64%) |  |  |
| Moderate | 60(41.67%) | 1,769(53.46%) | 2.72 (1.88-3.94, p<.001) | 2.32 (1.59-3.39, p<.001) |
| High | 24(16.67%) | 890(26.90%) | 3.42 (2.11-5.55, p<.001) | 2.76 (1.68-4.54, p<.001) |
| **Sociosexual orientation** |  |  |  |  |
| Unrestricted | 102(70.83%) | 1,615(48.81%) |  |  |
| Restricted | 42(29.17%) | 1,694(51.19%) | 2.55 (1.77-3.67, p<.001) | 2.15 (1.48-3.12, p<.001) |

## Supplementary Figures
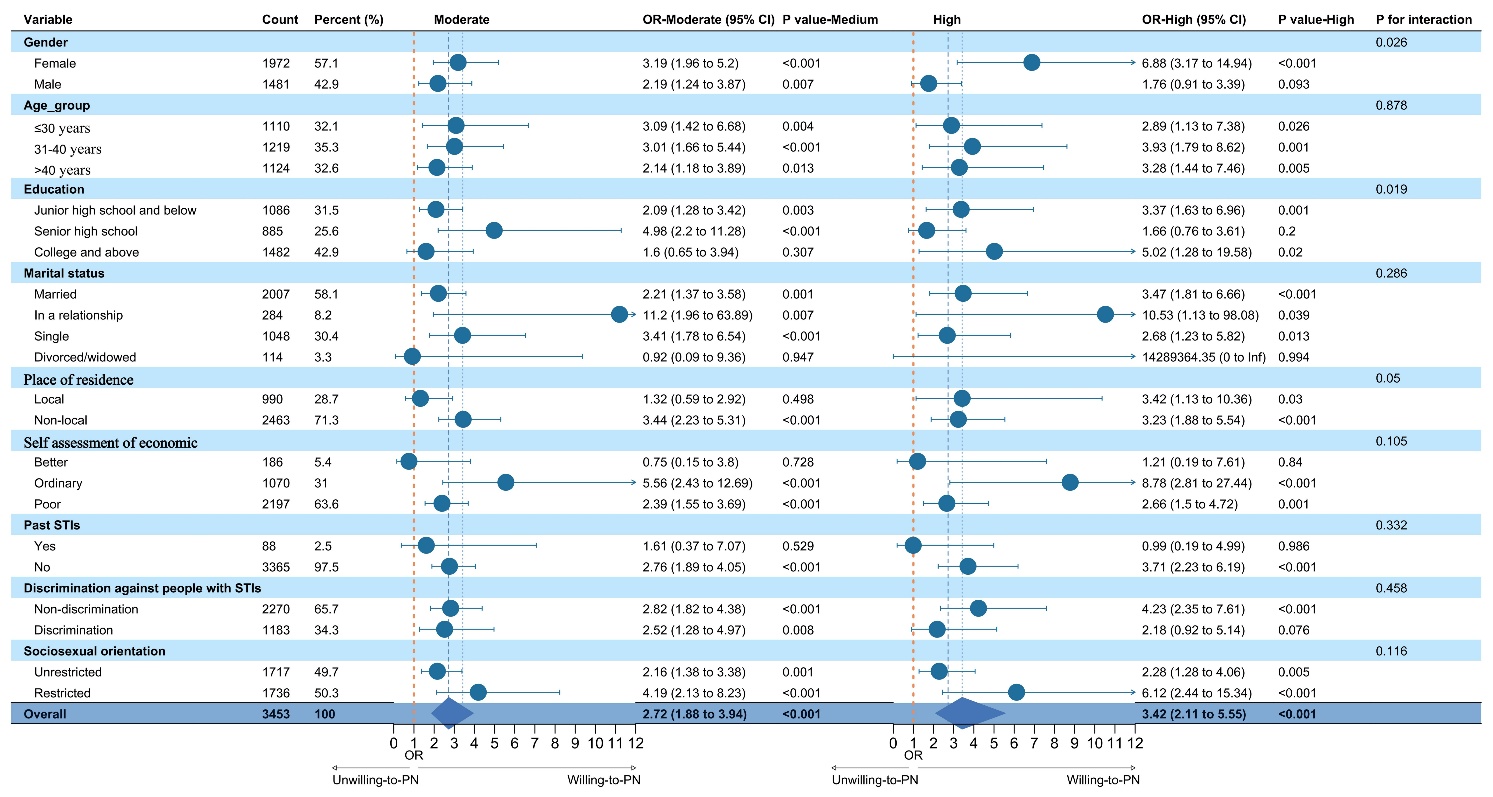


**Supplementary Figure 1.** Forest plot showing the association between perceived social support and PN willingness across demographic subgroups.
